# Supporting Newly Arrived Migrant Mothers: A Pilot Health Literacy Intervention

**DOI:** 10.3928/24748307-20210601-01

**Published:** 2021-07

**Authors:** Louise Dougherty, Annette Riley, Paula Caffrey, Alison Wallbank, Mary Milne, Mark F. Harris, Jane Lloyd

## Abstract

Experiencing migration can create or exacerbate vulnerability to ill health, particularly during pregnancy and new motherhood. Providing a culturally appropriate health literacy intervention to new migrant families may increase social support and the skills and confidence to access health care services and information. This study developed and piloted a health literacy intervention, in the form of culturally redesigned new parent classes, in a culturally diverse location in Australia. The intervention was delivered over a 4-week period by Child and Family Health Nurses, with the help of interpreters and Bilingual Community Researchers, to Bangladeshi and Mandarin-speaking Chinese mothers and grandmothers with a baby age 0 to 1 year. A mixed-methods evaluation was conducted to measure (1) recruitment and attendance of participants, (2) feasibility of the intervention, (3) health literacy of participants, and (4) provider understanding of barriers to health care access. Thirty participants were recruited, and 18 women attended at least three of the four group sessions. Nurses viewed the program as being within the scope of their usual role, demonstrating intervention feasibility. Health literacy scores were higher post-intervention than pre-intervention. Nurses described having increased awareness of barriers to health care access after facilitating the intervention. The program has potential to be scaled up to other areas and languages. **[*HLRP: Health Literacy Research and Practice*. 2021;5(3):e201–e207.]**

Migrant populations (i.e., those who have changed their country of residence, regardless of reason or legal status) (United Nations, 2020) face increased vulnerability to ill health and poor health outcomes for a number of reasons, including language and cultural barriers, socioeconomic status, stress, and structural barriers, all of which can limit access to health and social services (Keygnaert et al., 2016). This vulnerability may be compounded for migrant women during times of high health care needs, such as the period surrounding pregnancy and early motherhood (Almeida et al., 2013). During this period, migrant women have a higher incidence of poor maternal and neonatal outcomes, postpartum depression, and difficulty accessing services (Almeida et al., 2013). This is a particularly important issue in Australia given that more than one-quarter (28%) of the Australian population was born overseas (Australian Bureau of Statistics, 2018), which is double the population born overseas living in the United Kingdom (14.4%) (Rienzo & Vargas-Silva, 2018) and the United States of America (13.4%) (United States Census Bureau, 2019). Within some regions of Australia, such as Canterbury (which is in Sydney, New South Wales), almost one-half of the population was born overseas (48%) (Dowsett & Broome, 2018). One potential avenue to address barriers to accessing health care is through interventions aiming to improve health literacy. Most research has tended to focus on identifying these barriers rather than trialing interventions to overcome these barriers (Dougherty et al., 2020), and as such, this pilot study is an important contribution to the literature and to practice.

Health literacy can be defined as the set of knowledge, motivation, and competencies needed by people, families, and communities for understanding and managing health, including accessing health services (Sørensen et al., 2012). Migrants are more likely to have lower health literacy than non-migrant populations (Beauchamp et al., 2015; Berens et al., 2016; Villadsen et al., 2020; World Health Organization Regional Office for Europe, 2013). Low health literacy has been associated with greater risk of hospitalization, lower use of preventive services, and poorer health outcomes (Berkman et al., 2011; Bush et al., 2010).

This brief report describes the implementation and evaluation of a pilot health literacy intervention conducted in 2019 that aimed to increase dimensions of health literacy (specifically, knowledge and access to information), as well as situational determinants of health literacy (specifically, social support) (Sørensen et al., 2012), for new migrant mothers and grandmothers in the Canterbury region of Sydney (a part of Sydney Local Health District). The specific focus was on migrant Bangladeshi (i.e., Bangla-speaking) and Mandarin-speaking Chinese groups, as these two groups had the largest numbers of births in the Canterbury area (excluding English-speaking Australian mothers) prior to this study commencing (Canterbury Hospital, personal communication, August 10, 2018. The intervention consisted of (1) using Bilingual Community Researchers (BCRs) as a novel recruitment mechanism to reach new mothers and grandmothers who may not be connected to the health service and (2) delivering culturally redesigned new parent classes to this group with an interpreter.

## Methods

### Recruitment

Eligible mothers (Bangla- or Mandarin-speaking women who self-identified as a migrant, with a baby age 0 to 1 year, and who lived in Sydney Local Health District region) were recruited through two different channels: BCRs and Child and Family Health Nurses (CFHNs) (described below). In both recruitment methods, the grandmothers were also invited to attend the classes, given the important role that grandmothers often play in the early childhood period within both cultural groups.

BCRs are an emerging workforce who provide research support in languages other than English (Lloyd et al., 2019). Three female BCRs (two Bangla-speaking and one Mandarin-speaking) were employed by the study to identify eligible mothers through their networks and invite them to participate in the intervention. The BCRs had previously been involved in research studies and were employed to assist in this study because they shared the language and cultural identity of our target participants. They used their connections within the community to advertise and recruit new mothers to the program, including through the placement of flyers in locally significant locations (e.g., community centers, religious centers, medical centers, libraries).

CHFNs also invited eligible mothers to attend, usually during routine phone calls after women had given birth and during clinic or home visits. If the eligible mother was interested, the nursing staff asked the woman's permission to pass on her contact details to the BCR, who would then contact the mother to provide more information about the classes and to answer any questions in Bangla or Mandarin.

Both the BCRs and CFHNs received training on the recruitment process, which included the use of a recruitment script and the importance of emphasizing the voluntary nature of the program.

### Development and Delivery of Intervention

Session outlines for the culturally redesigned new parent classes were developed based on the local health service's existing new parent class outlines, with some cultural adaptation and prioritization of select content that occurred through a co-design workshop with Child and Family Health staff and the BCRs as community representatives. Four topics (one per week) were agreed upon as being the most relevant to focus on by both the Bangla- and Mandarin-speaking representatives. These topics are listed in **Table 1**, along with some examples of how these topics were modified from the existing local health service's new parent class outlines. Additional cultural adaptations included using an interpreter to deliver the classes in the participant's native language, holding the classes in suburbs where many of Sydney Local Health District's Bangla- and Mandarin-speaking community live, providing culturally relevant foods for afternoon tea, and the presence of the BCRs throughout all stages of the study. The session content was deliberately shorter than the allocated time-slot so that the participants could request any additional topics of interest to be covered by the CFHN (as well as to allow the participants to have time to make social connections).

**Table 1:**
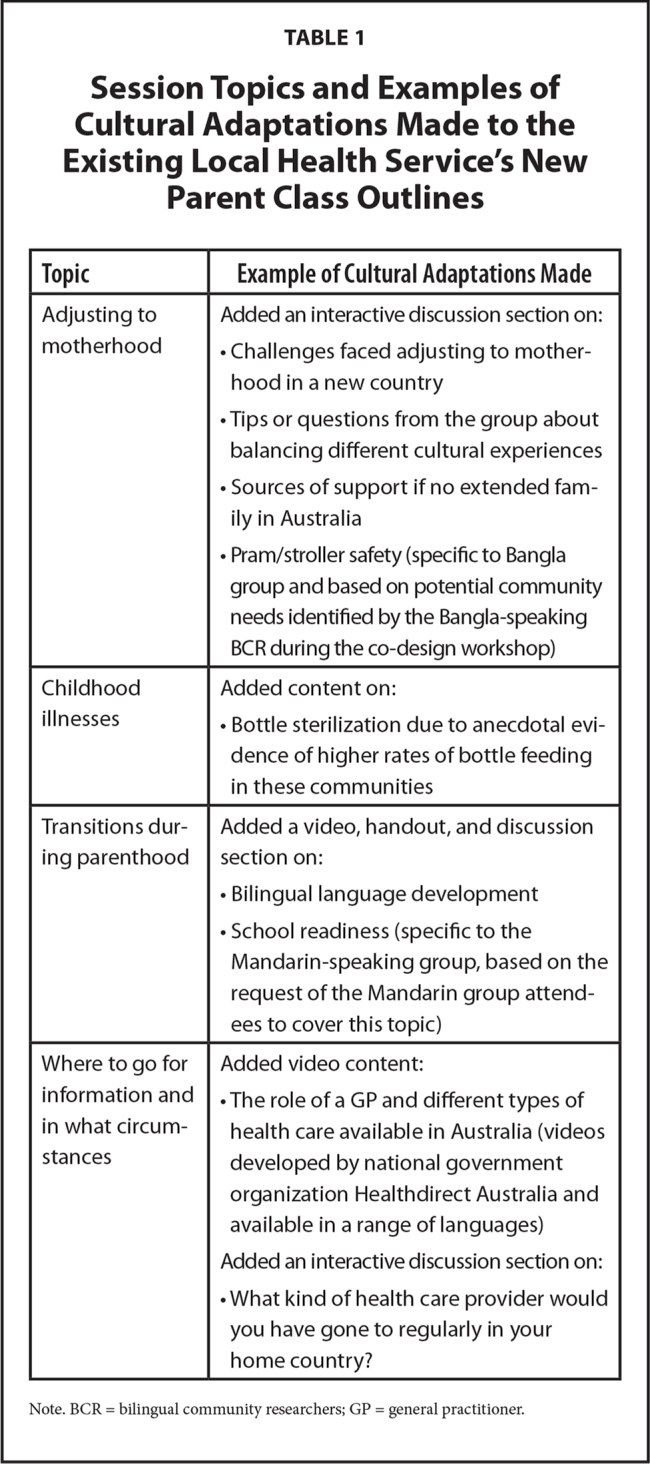
Session Topics and Examples of Cultural Adaptations Made to the Existing Local Health Service's New Parent Class Outlines

**Topic**	**Example of Cultural Adaptations Made**
Adjusting to motherhood	Added an interactive discussion section on: Challenges faced adjusting to motherhood in a new country Tips or questions from the group about balancing different cultural experiences Sources of support if no extended family in Australia Pram/stroller safety (specific to Bangla group and based on potential community needs identified by the Bangla-speaking BCR during the co-design workshop)
Childhood illnesses	Added content on: Bottle sterilization due to anecdotal evidence of higher rates of bottle feeding in these communities
Transitions during parenthood	Added a video, handout, and discussion section on: Bilingual language development School readiness (specific to the Mandarin-speaking group, based on the request of the Mandarin group attendees to cover this topic)
Where to go for information and in what circumstances	Added video content: The role of a GP and different types of health care available in Australia (videos developed by national government organization Healthdirect Australia and available in a range of languages) Added an interactive discussion section on: What kind of health care provider would you have gone to regularly in your home country?

Note. BCR = bilingual community researchers; GP = general practitioner.

The new parent classes were delivered to each language group once a week (in a 2-hour session) over a 4-week period at a local Child and Family Health Center. The sessions were delivered by a CFHN, with an interpreter. The BCRs contacted participants prior to the session to remind them to attend and helped to troubleshoot any issues for participants, such as how to get to the venue. The BCRs were also present at the sessions to support the participants as required.

### Evaluation of the Intervention

Process and outcome evaluation was conducted using a combination of qualitative and quantitative data collection techniques, including semi-structured interviews, focus groups, and questionnaires (**Table 2**).

**Table 2:**
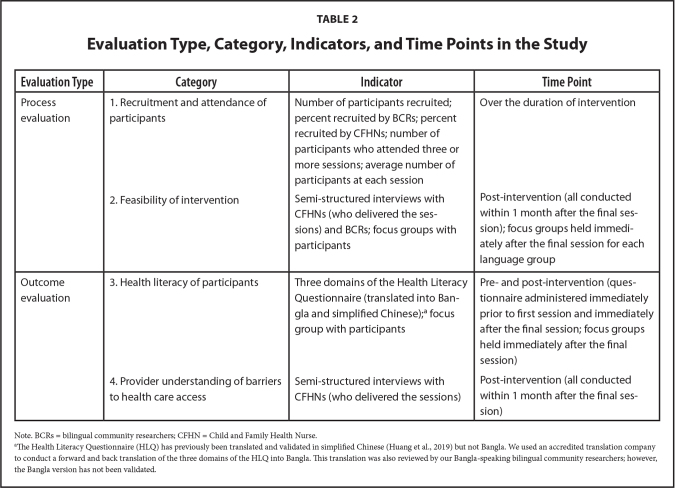
Evaluation Type, Category, Indicators, and Time Points in the Study

**Evaluation Type**	**Category**	**Indicator**	**Time Point**
Process evaluation	1. Recruitment and attendance of participants	Number of participants recruited; percent recruited by BCRs; percent recruited by CFHNs; number of participants who attended three or more sessions; average number of participants at each session	Over the duration of intervention
	2. Feasibility of intervention	Semi-structured interviews with CFHNs (who delivered the sessions) and BCRs; focus groups with participants	Post-intervention (all conducted within 1 month after the final session); focus groups held immediately after the final session for each language group

Outcome evaluation	3. Health literacy of participants	Three domains of the Health Literacy Questionnaire (translated into Bangla and simplified Chinese);^a^ focus group with participants	Pre- and post-intervention (questionnaire administered immediately prior to first session and immediately after the final session; focus groups held immediately after the final session)
	4. Provider understanding of barriers to health care access	Semi-structured interviews with CFHNs (who delivered the sessions)	Post-intervention (all conducted within 1 month after the final session)

Note. BCRs = bilingual community researchers; CFHN = Child and Family Health Nurse.

a The Health Literacy Questionnaire (HLQ) has previously been translated and validated in simplified Chinese (Huang et al., 2019) but not Bangla. We used an accredited translation company to conduct a forward and back translation of the three domains of the HLQ into Bangla. This translation was also reviewed by our Bangla-speaking bilingual community researchers; however, the Bangla version has not been validated.

Health literacy scores pre- and post- intervention were measured across three domains of the validated Health Literacy Questionnaire developed by Osborne, Batterham, Elsworth, Hawkins, and Buchbinder (2013). These are domain 2: “having sufficient information to manage health,” which contains four questions scored from one (lower) to four (higher); domain 4: “social support for health,” which contains five questions scored from one (lower) to four (higher); and domain 8: “ability to find good health information,” which contains five questions scored from one (lower) to five (higher) for both the Bangla- and Mandarin-speaking groups. As such, the Health Literacy Questionnaire provides separate scores for each domain measured, indicating a person's health literacy strengths and needs across different areas, rather than an overall summative score for all domains measured (Beauchamp et al., 2015). In total, the Health Literacy Questionnaire has nine domains (Osborne et al., 2013), however, only the three domains most closely matching our aims were measured, as these were the areas of health literacy that we sought to influence through this study. Health literacy scores were compared within and between groups, using descriptive statistics only due to the small sample size. Results were also compared with the findings of the Health Literacy Survey conducted by the Australian Bureau of Statistics in 2018, which was administered to a sample of 5,790 adults (Australian Bureau of Statistics, 2019).

The focus groups were led by the first author (L. D.) (with an interpreter), using a conversation guide. The semi-structured interviews with the BCRs and providers (CFHNs) were conducted by the research team (L. D. and J. L), using an interview guide. Both the focus groups and semi-structured interviews were recorded and transcribed. For the purposes of this pilot study, descriptive qualitative analysis was conducted. This method is relevant for studies where time and resources are limited (Bradshaw et al., 2017).

### Ethics

Evaluation activities for this project were approved by Sydney Local Health District (protocol number X18-0504; HREC/18/RPAH/724; SSA/19/RPAH/63) and ratified by the University of New South Wales Human Research Ethics Committee. Participants gave their voluntary signed consent to participate in the evaluation activities, with all study information sheets and consent forms provided in Bangla and Mandarin.

## Results

### Recruitment and Attendance

For the new-parent group who spoke Mandarin, a total of 16 participants were recruited (12 mothers and four grandparents). Of these, nine participants (seven mothers and two grandmothers) were recruited by BCRs, and seven participants (five mothers and two grandparents) were recruited by CFHNs. There were three mothers who registered for the group but did not attend any sessions [baby unwell (*n* = 1); family matter (*n* = 1); unknown reason (*n* = 1)]. Thirteen participants (nine mothers and four grandparents) attended at least one session. Attendance was greater for participants who were recruited by BCRs than those recruited by CFHNs, with seven of 10 (70%) of the participants who attended three or more of the four sessions having been recruited by BCRs.

For the classes with Bangla-speaking participants, a total of 14 were recruited (12 mothers and two grandmothers). Of these, 12 participants (10 mothers and two grandmothers) were recruited by BCRs, and two participants (two mothers) were recruited by CFHNs. There were two mothers who registered for the group but did not attend any sessions (for unknown reasons). Twelve participants (10 mothers and two grandmothers) attended at least one session. Again, attendance was greater for the participants who were recruited via BCR, with seven of the eight participants who attended three or more sessions having been recruited by BCRs (87.5%).

### Feasibility

The groups were considered to be feasible based on the qualitative feedback received from the interviews with the BCRs (*n* = 3), CFHNs (*n* = 2), and the focus groups with participants (Bangla group: *n* = 12; Mandarin group: *n* = 10). The CFHNs were comfortable using an interpreter and considered the classes delivered as part of this pilot study to be broadly in keeping with their usual practice. The attendees were interested in attending more groups in the future to further expand their knowledge of children's health. The BCRs highlighted that running courses with the nurse speaking in English and the interpreter translating into Bangla or Mandarin provided an opportunity for strengthening the English ability of the attendees while also ensuring that the sessions were accessible to mothers with limited English skills.

### Health Literacy

**Figure 1.**
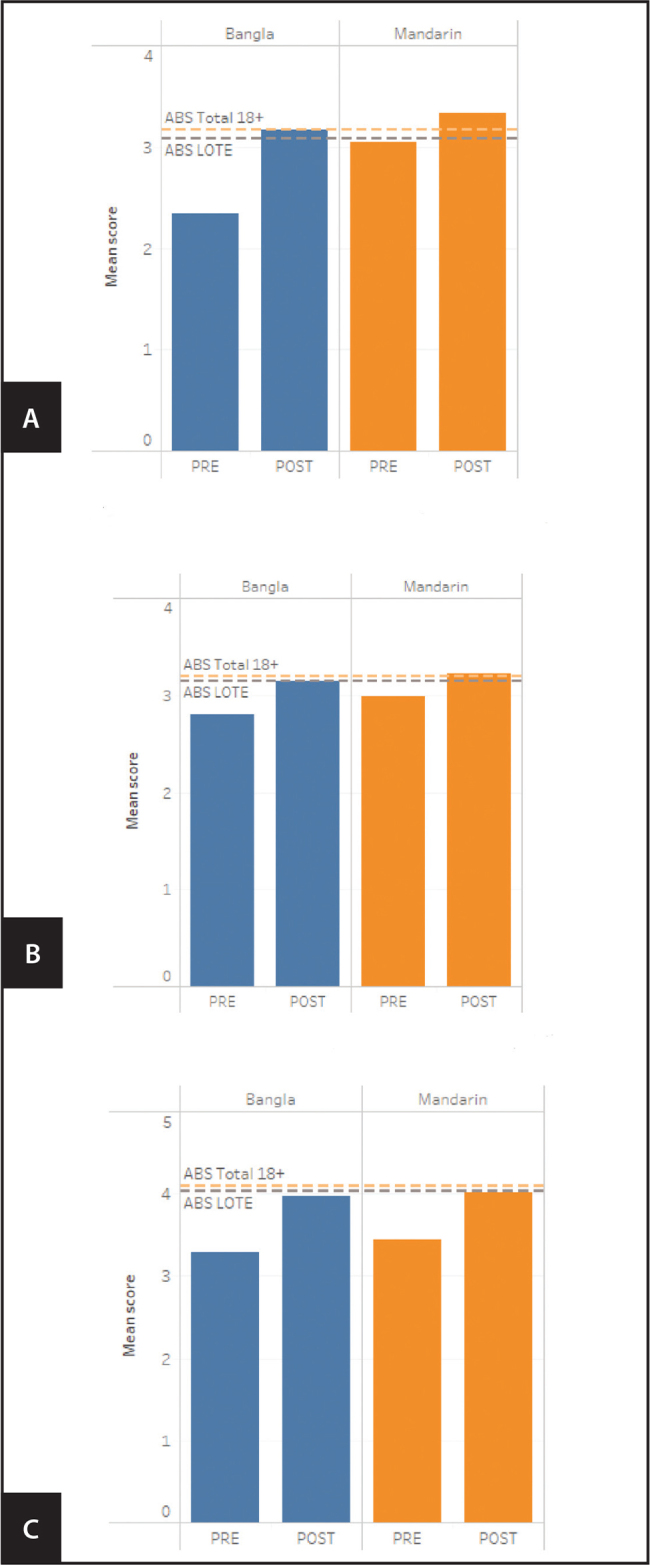
Pre- and post-intervention scores for three domains of the Health Literacy Questionnaire, with reference lines showing average scores from a sample of Australians (Australian Bureau of Statistics [ABS] Total 18+) and a sample of Australians who speak a language other than English at home (LOTE). (A) Domain 2: “Have sufficient information to manage health” (scored out of 4). (B) Domain 4: “Social support for health” (scored out of 4). (C) Domain 8: “Ability to find good health information” (scored out of 5).

Data were analyzed for those who attended three or more of the four sessions, which was 77% (10/13) of the Mandarin-speaking group and 67% (8/12) of the Bangla-speaking group. **Figure 1** demonstrates how these scores compare to the average score from a sample of Australians (age 18 years and older) surveyed by the Australian Bureau of Statistics as well as the average score from the sample of Australians who speak a language other than English (LOTE) at home in the Australian Bureau of Statistics dataset (Australian Bureau of Statistics, 2019).

Both the Bangla- and Mandarin-speaking groups had higher health literacy across all three health literacy domains at the post-intervention survey compared to the pre-intervention survey (**Figure 1**). For domain 2 (**Figure 1A**), both groups equaled or exceeded both Australian Bureau of Statistics (ABS) averages at the post-intervention time point. The Bangla-speaking group had a lower pre-intervention mean score than the Mandarin-speaking group. For domain 4 (**Figure 1B**), both groups equaled or exceeded the average score from the ABS sample of Australians who speak a LOTE at home at the post-intervention time point. The Mandarin-speaking group also exceeded the average score for the total ABS sample of adults age 18 years and older at the post-intervention time point by a small margin. For domain 8 (**Figure 1C**), by the post-intervention time point, both groups had moved closer to the average scores identified during the ABS survey but were still slightly below the average for both Australians age 18 years and older and Australians who speak a LOTE at home.

### Provider Understanding of Barriers

As a result of being involved in the pilot project, CFHNs described having increased cultural understanding of the experiences and needs of new mothers and grandmothers who are migrants, as well as awareness of a number of potential barriers to health care access, including lack of awareness from other health care providers about the Child and Family Health Service. It was identified that having closer links with primary care practice managers or practice nurses may be a useful strategy to promote their services.

## Discussion

A recent literature review has identified that there are a lack of evaluated interventions to improve health literacy and health care access for migrant families who speak a language other than English, with the majority of the literature focused on identifying enablers and barriers to health care access rather than testing interventions (Dougherty et al., 2020). However, there is a clear need for these types of interventions given that migrant women have been found to experience lower levels of access to health care and poorer birth outcomes (Almeida et al., 2013). The results of this pilot study suggest that this type of intervention may be a feasible mechanism to improve aspects of health literacy for migrant mothers and grandmothers and increase provider understanding of barriers to health care access for these groups.

The BCRs played an important role throughout the project by informing the development of the intervention, recruiting women to the intervention, and providing cultural support during the delivery of the intervention. The connections and trust of the BCRs within their communities may have been a key contributing factor to the success of the project. Other studies have highlighted the important role that bilingual workers can play in improving knowledge and awareness of health services (Henderson et al., 2011), as well as in improving recruitment processes and overall intervention effectiveness as a result of their cultural understanding and sensitivity (Krok-Schoen et al., 2016).

Working in a collaborative partnership with CFHNs to deliver the intervention enabled providers to gain a deeper understanding of some of the issues that might affect access to health care for new migrants and led to increased cultural knowledge and awareness. Feasibility aspects such as providers being able to deliver the intervention within the scope of their normal role provide a strong case for the scalability of this intervention to include other areas and language groups.

## Study Limitations

There are some limitations to note when considering the results of this pilot study. First, the Health Literacy Questionnaire was translated into Bangla for this study but has not yet been validated for this population. As such, the health literacy scores of the Bangla-speaking participants need to be interpreted with caution. Second, this pilot study only had a small sample size, and as a result, we are unable to draw robust, statistical conclusions about the effectiveness of the intervention. However, the results can be interpreted with caution to demonstrate that this intervention may have had a positive effect on health literacy. Further studies with a larger sample size and a control group should be conducted by researchers and health care services to provide higher quality evidence of the impact of this type of intervention.

## References

[x24748307-20210601-01-bibr1] Almeida, L. M., Caldas, J., Ayres-de-Campos, D., Salcedo-Barrientos, D., & Dias, S. (2013). Maternal healthcare in migrants: A systematic review. *Maternal and Child Health Journal*, *17*(8), 1346–1354. 10.1007/s10995-012-1149-x PMID:23334357

[x24748307-20210601-01-bibr2] Australian Bureau of Statistics. (2018). *Australia's population by country of birth - 3412.0 - Migration, Australia, 2016–17*. https://www.abs.gov.au/AUSSTATS/abs@.nsf/Lookup/3412.0Main+Features32016-17?OpenDocument

[x24748307-20210601-01-bibr3] Australian Bureau of Statistics. (2019). *National health survey: Health literacy, 2018*. https://www.abs.gov.au/AUSSTATS/abs@.nsf/Lookup/4364.0.55.014Main+Features12018?OpenDocument

[x24748307-20210601-01-bibr4] Beauchamp, A., Buchbinder, R., Dodson, S., Batterham, R. W., Els-worth, G. R., McPhee, C., Sparkes, L., Hawkins, M., & Osborne, R. H. (2015). Distribution of health literacy strengths and weaknesses across socio-demographic groups: A cross-sectional survey using the Health Literacy Questionnaire (HLQ). *BMC Public Health*, *15*, 678. 10.1186/s12889-015-2056-z PMID:26194350 PMC4508810

[x24748307-20210601-01-bibr5] Berens, E.-M., Vogt, D., Messer, M., Hurrelmann, K., & Schaeffer, D. (2016). Health literacy among different age groups in Germany: Results of a cross-sectional survey. *BMC Public Health*, *16*(1), 1151. 10.1186/s12889-016-3810-6 PMID:27829401 PMC5103460

[x24748307-20210601-01-bibr6] Berkman, N., Sheridan, S., Donahue, K., Halpern, D., Viera, A., Crotty, K., Holland, A., Brasure, M., Lohr, K. N., Harden, E., Tant, E., Wallace, I., Viswanathan, M. (2011). *Health literacy interventions and outcomes: An updated systematic review*. Agency for Healthcare Research and Quality. https://www.ahrq.gov/downloads/pub/evidence/pdf/literacy/literacyup.pdf PMC4781058 23126607

[x24748307-20210601-01-bibr7] Bradshaw, C., Atkinson, S., & Doody, O. (2017). Employing a qualitative description approach in health care research. *Global Qualitative Nursing Research*, *4*, 1–8. 10.1177/2333393617742282 PMC5703087 29204457

[x24748307-20210601-01-bibr8] Bush, R., Boyle, F., Ostini, R., Ozolins, I., Brabant, M., Soto, E. J., & Eriksson, L. (2010). *Advancing health literacy through primary health care systems*. Australian National University College of Health and Medicine. https://openresearch-repository.anu.edu.au/bit-stream/1885/119189/1/Bush_Health_literacy_final_report.pdf

[x24748307-20210601-01-bibr9] Dougherty, L., Lloyd, J., Harris, E., Caffrey, P., & Harris, M. (2020). Access to appropriate health care for non-English speaking migrant families with a newborn/young child: A systematic scoping literature review. *BMC Health Services Research*, *20*(1), 309. 10.1186/s12913-020-05157-x 32293440 PMC7158115

[x24748307-20210601-01-bibr10] Dowsett, M., & Broome, R. (2018). *A demographic and social profile of Sydney Local Health District*. New South Wales Government. https://www.slhd.nsw.gov.au/PopulationHealth/pdf/slhd_demography_2016.pdf

[x24748307-20210601-01-bibr11] Henderson, S., Kendall, E., & See, L. (2011). The effectiveness of culturally appropriate interventions to manage or prevent chronic disease in culturally and linguistically diverse communities: A systematic literature review. *Health & Social Care in the Community*, *19*(3), 225–249. 10.1111/j.1365-2524.2010.00972.x 21208326

[x24748307-20210601-01-bibr12] Huang, Y., Ruan, T., Yi, Q., Wang, T., & Guo, Z. (2019). The health literacy questionnaire among the aged in Changsha, China: Confirmatory factor analysis. *BMC Public Health*, *19*(1), 1220. 10.1186/s12889-019-7563-x PMID:31484512 PMC6727331

[x24748307-20210601-01-bibr13] Keygnaert, I., OIvanova, O., Guieu, A., Van Parys, A.-S., Leye, E., & Roelens, K. (2016). *What is the evidence on the reduction of inequalities in accessibility and quality of maternal health care delivery for migrants? A review of the existing evidence in the WHO European Region*. World Health Organization. http://www.euro.who.int/__data/assets/pdf_file/0003/317109/HEN-synthesis-report-45.pdf?ua=1 27786434

[x24748307-20210601-01-bibr14] Krok-Schoen, J. L., Weier, R. C., Hohl, S. D., Thompson, B., & Paskett, E. D. (2016). Involving community health workers in the centers for population health and health disparities research projects: Benefits and challenges. *Journal of Health Care for the Poor and Underserved*, *27*(3), 1252–1266. 10.1353/hpu.2016.0145 PMID:27524766 PMC8856581

[x24748307-20210601-01-bibr15] Lloyd, J., Dougherty, L., Dennis, S., Attenbrow, H., Harris, E., Wise, M., & Harris, M. (2019). Culturally diverse patient experiences and walking interviews: A co-design approach to improving organizational health literacy. *HLRP: Health Literacy Research and Practice*, *3*(4), e238–e242. 10.3928/24748307-20190828-01 PMID:31637364 PMC6786687

[x24748307-20210601-01-bibr16] Osborne, R. H., Batterham, R. W., Elsworth, G. R., Hawkins, M., & Buchbinder, R. (2013). The grounded psychometric development and initial validation of the Health Literacy Questionnaire (HLQ). *BMC Public Health*, *13*(1), 658. 10.1186/1471-2458-13-658 PMID:23855504 PMC3718659

[x24748307-20210601-01-bibr17] Rienzo, C., & Vargas-Silva, C. (2018). *Migrants in the UK: An overview*. The Migration Observatory. https://migrationobservatory.ox.ac.uk/resources/briefings/migrants-in-the-uk-an-overview/

[x24748307-20210601-01-bibr18] Sørensen, K., Van den Broucke, S., Fullam, J., Doyle, G., Pelikan, J., Slonska, Z., Brand H.; the (HLS-EU) Consortium Health Literacy Project European. (2012). Health literacy and public health: A systematic review and integration of definitions and models. *BMC Public Health*, *12*(1), 80. 10.1186/1471-2458-12-80 PMID:22276600 PMC3292515

[x24748307-20210601-01-bibr19] United Nations. (2020). *Refugees and migrants*. https://refugeesmigrants.un.org/definitions

[x24748307-20210601-01-bibr20] United States Census Bureau. (2019). *Quick facts*. https://www.census.gov/quickfacts/fact/table/US/PST045218

[x24748307-20210601-01-bibr21] Villadsen, S. F., Hadi, H., Ismail, I., Osborne, R. H., Ekstrøm, C. T., & Kayser, L. (2020). eHealth literacy and health literacy among immigrants and their descendants compared with women of Danish origin: A cross-sectional study using a multidimensional approach among pregnant women. *BMJ Open*, *10*(5), e037076–e037076. 10.1136/bmjopen-2020-037076 PMID:32385065 PMC7228522

[x24748307-20210601-01-bibr22] World Health Organization Regional Office for Europe. (2013). *Health literacy: The solid facts*. https://apps.who.int/iris/handle/10665/326432

